# Exploring Carbon Nanomaterial Diversity for Nucleation of Protein Crystals

**DOI:** 10.1038/srep20053

**Published:** 2016-02-04

**Authors:** Lata Govada, Hannah S. Leese, Emmanuel Saridakis, Sean Kassen, Benny Chain, Sahir Khurshid, Robert Menzel, Sheng Hu, Milo S. P. Shaffer, Naomi E. Chayen

**Affiliations:** 1 Computational and Systems Medicine, Department of Surgery and Cancer, Faculty of Medicine, Imperial College London, London SW7 2AZ, UK; 2 Department of Chemistry, Imperial College London, London SW7 2AZ, UK; 3 Laboratory of Structural and Supramolecular Chemistry, Institute of Nanoscience and Nanotechnology, National Centre for Scientific Research ‘Demokritos’ Athens, Greece; 4 Division of infection and immunity, The Cruciform Building, UCL, Gower St., London WC1E 6BT

## Abstract

Controlling crystal nucleation is a crucial step in obtaining high quality protein crystals for structure determination by X-ray crystallography. Carbon nanomaterials (CNMs) including carbon nanotubes, graphene oxide, and carbon black provide a range of surface topographies, porosities and length scales; functionalisation with two different approaches, gas phase radical grafting and liquid phase reductive grafting, provide routes to a range of oligomer functionalised products. These grafted materials, combined with a range of controls, were used in a large-scale assessment of the effectiveness for protein crystal nucleation of 20 different carbon nanomaterials on five proteins. This study has allowed a direct comparison of the key characteristics of carbon-based nucleants: appropriate surface chemistry, porosity and/or roughness are required. The most effective solid system tested in this study, carbon black nanoparticles functionalised with poly(ethylene glycol) methyl ether of mean molecular weight 5000, provides a novel highly effective nucleant, that was able to induce crystal nucleation of four out of the five proteins tested at metastable conditions.

X-ray crystallography is to date the most successful and prolific method for the determination of protein and nucleic acid three-dimensional structures at high resolution. Obtaining the necessary high quality macromolecular crystals has for decades been the major bottleneck to the advance of both biological science and medicine. Therefore, the search for substances that can effectively induce the nucleation of crystals is a very active and promising endeavour. The nucleation of protein crystals using heterogeneous materials was first reported twenty five years ago; McPherson and Shlichta tested over fifty mineral substrates as nucleating agents on which protein crystals grew epitaxially^1^. Despite the promising concept, the selected materials had limited success, prompting the quest for additional nucleating agents. A new approach, in 2001, presented the idea of using porous materials^2^ as nucleating agents; it is hypothesised that nucleation is induced by entrapping protein molecules in the pores, thus encouraging them to form crystalline aggregates without involving an epitaxial mechanism. A host of other porous nucleants with pore sizes on the order of magnitude of protein molecules have since been designed, developed and tested; Bioglass^3^ (commercially available as ‘Naomi’s Nucleant’), nanoporous gold and microporous zeolite^4^ are some examples. Other candidate substances such as Sephadex beads, mica^5^, human^6^ and horse hair^7^,^8^, seaweed^8^, charged surfaces^9^, and protein thin films^10^, have been successful on a number of proteins but have not yet been adopted for general use. One important reason is that such substances are difficult either to prepare or to introduce systematically into crystallization trials. Recent theoretical studies have modelled the heterogeneous nucleation mechanism, and hypothesised that geometry (i.e. the presence of pores within a range of sizes, roughness and/or microstructured surfaces) critically controls protein nucleation^3^,^11^,^12^,^13^,^14^.

This study tests the efficacy of a variety of functionalised and non-functionalised carbon nanomaterials (CNMs) as nucleating agents. The focus is on carbon nanotubes of various dimensions, but with comparison to graphite, graphene oxide (GO) and carbon black (CB). CNMs have attracted significant interest due to their outstanding intrinsic properties (structural, mechanical and electrical)^15^,^16^ and their potential for a broad range of applications in engineering, bio-nanotechnology, and catalysis. As discussed in more detail below, their characteristics may also be relevant to the nucleation challenge; in addition, the panel of materials selected provides a means to systematically compare the effects of surface chemistry through functionalisation and material length scale. The only previously published work on the use of carbon nanotube materials as potential nucleants for protein crystallization was by Asanithi *et al*.^17^, who tested “Buckypaper” – films of entangled carbon nanotubes. Colloidal graphene and graphene oxide have also been shown to lead to (limited) induction of crystal nucleation in certain cases^18^. Networks of carbon nanotubes and stacks of graphene sheets present a large surface heterogeneity with a number of differently sized, inter-particle ‘pockets’ in which protein crystals may be able to nucleate. A particularly interesting possibility compared to previous work on porous and microstructured materials as nucleants, is the opportunity for chemical modification, which can enhance the heterogeneous topography. The interactions of the protein with highly accessible surface areas and extensive porosity can be modulated through functionalisation.

Carbon nanotubes (CNTs) are broadly graphitic cylindrical structures with a wide range of diameters (~0.5–100 nm), lengths (~0.1–20 μm), crystallinity, and curvature, depending on synthesis route^19^. Multi-walled carbon nanotubes (MWNTs) may be particularly interesting substrates for protein nucleation due to their high aspect ratios and tendency to form robust open networks with very high accessible surface areas and large porosity. The influence of surface curvature is an interesting open question in nucleation. In principle, the absolute curvature of CNTs is controlled by their diameters; in addition, both positive (external tube surface) and negative (internal surface of opened CNTs) may be available.

The most common chemical vapour deposition (CVD)-grown CNTs generally have oxide defects on the surface which can be exploited for further functionalisation^20^,^21^, without inducing any further damage or alteration of the structure. A comparable catalogue of functionalised materials can, therefore, be created which are useful for many applications. In this study, the CNT surfaces are functionalised with different oligomers thus changing the surface chemistry, solvent wettability, and presumably protein interaction. Functionalised CNTs are easier to disperse and form into consistent networks (Fig. 1a,b). This study has mainly focused on functionalised MWNTs through a thermochemical grafting method^20^,^21^,^22^. However, a second method utilising reduction chemistry^23^,^24^,^25^,^26^ can also be used to functionalise other nanocarbon geometries, since the gas phase approach only modifies accessible surface area. This approach can lead to spontaneous exfoliation of charged species for improved functionalisation of individualised nanocarbons. Polyethylene glycol (PEG) is of particular interest, due to its use in protein crystallization as a precipitating agent and additive. Carbon black (CB) forms different sized aggregates made of 10 – 20 nm primary nanoparticles (Fig. 1c,d).

In all, the nucleation effectiveness of 20 different CNMs, varied by carbon substrate and/or functionalisation (Tables 1 and 2), was tested by introducing small solid flakes or chips of each into solutions containing protein, buffer and precipitating agent, at metastable conditions. These conditions, different for each of the proteins (see Experimental Section), are normally able to sustain the growth of protein crystals but correspond to supersaturations that are not sufficient for crystals to nucleate. Five different proteins were tested against each CNM. Four are often used as standards or models for crystallization studies^27^; they are thaumatin, trypsin, lysozyme and catalase. The fifth was a target protein, antibody RoAb13, the structure of which was recently solved^28^. The ability of each CNM to induce nucleation was assessed by the presence of crystals in the metastable drops.

## Results

By utilising the diverse nature of the carbon nanomaterial family, it is possible to explore these materials systematically as nucleants in their as-received and functionalised forms. Table 1 summarises the nanocarbon substrates, which provide a wide range of surface areas, geometries and sizes ; graphite (BET < 4 m^2^ g^-1^) was also used as a control. The versatility of the thermochemical grafting method allowed functionalisation of MWNTs with the monomers shown in Table 2. The range of oligomers is wide, and includes anionic, cationic, and non-ionic moieties, as well as mixtures of hydrophilic and hydrophobic species; different functional groups (such as thioether, amines, and hydroxyl) may also affect specific interactions with proteins. The detailed synthesis and characterisation of these new materials is described in references provided in Table 2; here, they are referred to by carbon nanomaterial acronym (CNM) and number (i.e. CNM 1 refers to ungrafted MWNTs).

The 20 CNMs (Table 2) used in this work were introduced into crystallization trials set at metastable conditions for each of the five proteins. In this study, metastable conditions were defined as conditions where all or almost all of a great number (at least three at each concentration) of control drops (left undisturbed and in the absence of any nucleation-inducing agent) had remained clear for at least one week after set up. All trials were set up using the vapour diffusion technique, except for lysozyme, where microbatch experiments were also performed (see below and in the Experimental Section). The results are summarised in Table 3 and described in greater detail below. Borderline effectiveness refers to cases where a small number of controls at the same conditions also yielded crystals albeit after a longer time, or the conditions at which the CNMs induced nucleation were only at slightly lower supersaturations than conditions that would also produce crystals in their absence. Strong effectiveness refers to cases where none of the corresponding controls yielded crystals and to cases where nucleation was induced at substantially lower supersaturations than the conditions that would yield crystals without the nucleant being present.

As discussed, PEG is already used in many protein crystallization conditions; therefore, it was important to make sure that the PEGylated nucleants were effective due to the immobilised PEG chains on the carbon nanomaterials rather than simply the introduction of additional PEG. Several controls were studied in which between 0.1 and 1 w/v% PEG of mean molecular weight 5000 (PEG 5 K) was added to the protein crystallization trials and observed over several days (up to 28 days). No crystals were observed, confirming that the grafted nanomaterial is the active nucleant in subsequent trials.

### Thaumatin

Solutions of 30 mg/ml protein containing 0.20 M NaK tartrate (NaKT) were found to be metastable. Controls at 0.25 M NaKT also remained clear in the large majority of the controls.

Trials containing CNMs 4, 6, 8, 10 and 20 yielded crystals at metastable conditions within a week after setup. Of these, CNMs 8 and 10 acted quickest, yielding crystals within 24 hours. CNMs 3, 9, 11, 12, 15, 17 and 18 accelerated nucleation, producing crystals after 24 hours at the borderline conditions of 0.25 M NaKT.

### Lysozyme

Solutions of 20 mg/ml protein containing 0.43 M sodium chloride were found to be metastable.

Crystals were obtained within 10 days at these conditions only in trials containing CNM 8. Other CNMs that gave crystals at borderline conditions (where most, but not all controls had remained clear) were CNM 4 (at 0.44 M NaCl) and CNM 1 (at 0.46 M NaCl). As these vapour diffusion results were rather limited, they were complemented by a series of similar experiments, set up using the microbatch technique. Metastable conditions extended down to 0.40 M NaCl, at which conditions CNM 20 yielded crystals within 24 hours, CNMs 11, 18 and 20 within 48 hours and CNM 13 after one week. Controls remained clear for at least 9 days after setup.

### Trypsin

Solutions of 60 mg/ml protein with between 16 and 18%(w/v) PEG of mean molecular weight 8000 (PEG 8 K) were found to be metastable.

**Figure 2:**
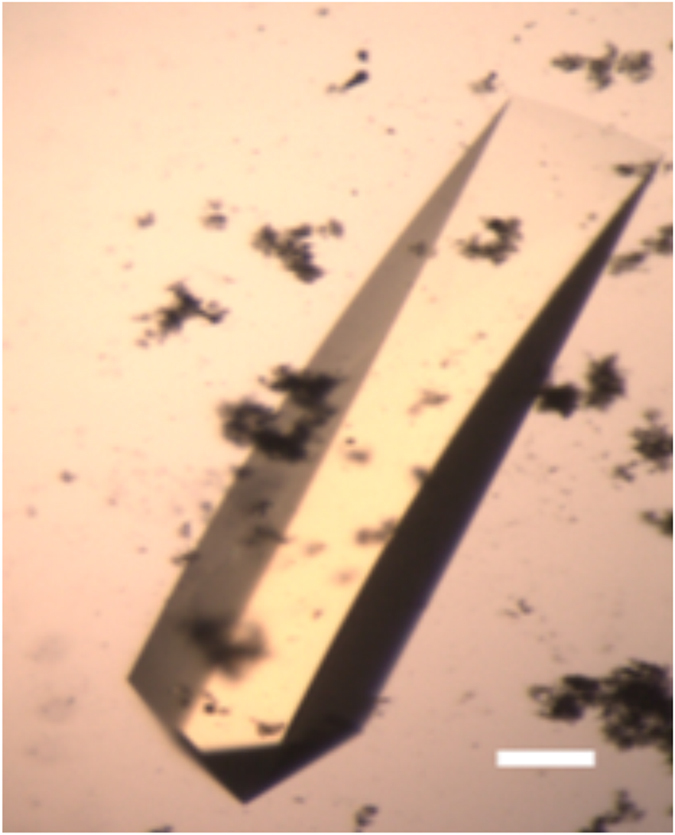
Trypsin crystals grown on CNM 20 deep in the metastable zone. Scale bar corresponds to 50 μm. The nucleant can be seen as black flakes.

CNMs 18 and 20 produced crystals throughout the metastable zone within 7 days from setup. Of these, CNM 20 was the most effective, as it produced crystals at all three PEG concentrations after 24 hours, (Fig. 2) while all other trials still remained clear. CNMs 5 and 12 were less effective, yielding crystals only at 18% PEG 8 K.

### Catalase

Solutions of 15 mg/ml protein with between 12 and 13%(w/v) of the primary precipitant, PEG of mean molecular weight 3350 (PEG 3350), were found to be metastable. 14%(w/v) PEG 3350 was a borderline condition where most, but not all controls remained clear for at least one week after setup.

Trials containing CNM 6 produced crystals at 13% PEG 3350 within one week. CNM 11 gave crystals at 14% PEG 3350 after 24 hours. CNMs 9 and 15 also gave crystals at 14% PEG 3350 after one week.

### RoAb13

Solutions of 10 mg/ml protein with between 17 and 19%(w/v) of the primary precipitant, PEG methyl ether of mean molecular weight 2000 (mPEG 2 K), were found to be metastable.

**Figure 3:**
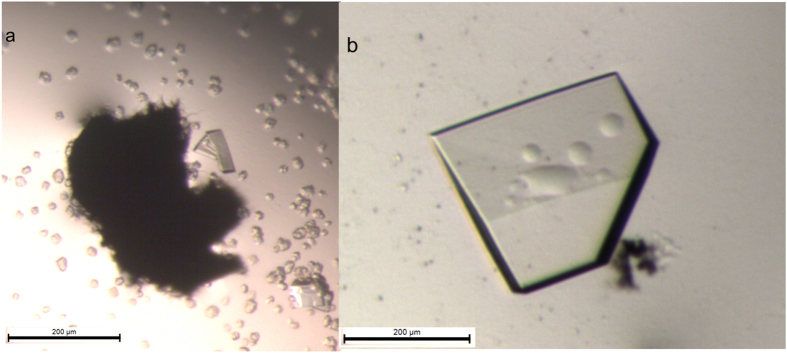
RoAb13 crystals grown on different CNMs. (**a**) small crystals on CNM 16 grown at the cusp of metastable and nucleation conditions (no crystals grew in metastable conditions). (**b**) single crystal grown on CNM 20 deep in the metastable zone. The nucleant is seen as black flakes.

Crystals were obtained from CNM 20 in 17 and 18% mPEG 2 K trials within 10 days from setup. A single crystal was observed with the 17% mPEG 2 K condition. Solutions containing 19% mPEG 2 K gave crystals with all CNMs, so these borderline cases were not taken into account (Fig. 3a).

An interesting phenomenon occurred in the case of RoAb13: initially crystals of RoAb13 were obtained both with and without nucleant. After a period of 12 months when trying to reproduce RoAb13 crystals without the nucleant, for another study, no crystals could be obtained at all despite repeated trials. It was feared that the protein had become inactive and that the project lost. The introduction of CNM 20 into the trials, however, gave good crystals thereby reviving the project (Fig. 3b).

In terms of the solid nucleants:

### Summary of CNMs

**CNM 20** (CB mPEG 5 K) was found to be the most potent nucleant in this study, as it was clearly effective with three model proteins (trypsin, thaumatin, lysozyme) and the antibody RoAb13. Furthermore, (i) it induced nucleation of lysozyme crystals quicker than any other effective CNM and (ii) it remained effective in nucleating RoAb13 crystals at lower supersaturations than any other CNM.

**CNM 18** (GO) was less effective, with two proteins (lysozyme, trypsin) for which it induced crystal nucleation in the metastable zone and one (thaumatin) where it promoted nucleation at borderline metastable conditions.

**CNMs 6 and 8** (functionalised MWNTs) came next in order of effectiveness, inducing nucleation at metastable conditions for two proteins each (thaumatin and catalase for CNM 6, thaumatin and lysozyme for CNM 8).

**CNMs 4, 10, 11, 13** (functionalised MWNTs) proved effective for only one protein each (thaumatin for 4 and 10; lysozyme for 11, 13). From those, CNM 11 also had a marginal nucleation inducing effect for thaumatin and catalase, and CNM 4 for lysozyme.

Several of the other candidate nucleants in this study also had some effect, but only at borderline conditions.

Therefore, the most effective nucleant tested was carbon black grafted with PEG 5 K, the second best being graphene oxide. Below these in order of effectiveness, came two MWNTs (A), one grafted with MTEMA and the other acid oxidised but ungrafted. Below these, effective only for one test protein each, came several more MWNTs (A) and the unreacted materials. The protein crystallization trials were repeated at least three times and consistently yielded the same result.

## Discussion

A new, highly effective custom-made solid nucleant was found: carbon black functionalised with mPEG 5 K. It rapidly induced nucleation in the metastable zone for 4 out of the 5 proteins with which the 20 candidate nucleants were tested. It was the only effective nucleant tested for one of these 4 proteins (RoAb13) and the most effective for another (lysozyme).

This study, as it involved a large number of related but diverse materials, also allows some general observations on the parameters that may influence heterogeneous nucleation. Various effects are thought to possibly play a role in facilitating nucleation of macromolecular crystals, namely effects due to electrostatic attraction from charges on the surfaces of protein and nucleant, and effects due to porosity, roughness or microstructure of the nucleant surface that may lead to confinement of the protein molecules. In many cases, changes in chemistry and structure may not be independent, since in a solid nucleant, packing between the particles determines pore size and is controlled partly by chemistry.

### Surface charge and functionalities

Based on their theoretical isoelectric points (IEP) (as obtained from the ExPASy server, http://web.expasy.org/compute_pi/), all the proteins tested have a net positive charge at their crystallization pH, except catalase which is neutral. The most successful nucleant CNM 20 has a net small negative surface charge at pH 7, although the PEG chain itself is neutral^22^. CNM 6 is likely to be similarly charged in the crystallization conditions. Graphene oxide (CNM 18) is also negatively charged (see Table 2 for available isoelectric point (IEP) values for all CNMs)^29^, except at the lysozyme crystallization pH at which it is neutral. The acid oxidised ungrafted MWNT(A) (CNM 8) are negative in all cases^21^. Both GO and the acid oxidised MWNTs were relatively successful in protein crystallization suggesting a positive effect when the surface charge and protein have opposite charges^30^. Same charge nucleant-protein combinations were much less successful, with only one moderately successful combination of highly positively charged lysozyme with positively charged MeVP-grafted MWNT(A), with a zeta potential of approximately +10 mV (CNM 13, Table 2). The success of same charge nucleant-protein crystallization is attributed to local, specific electrostatic effects which can be more important than the non-specific attraction due to opposite net charges (a proposal that has been put forward by Fermani *et al*.^31^ and by Liu *et al*.^32^ to explain cases of nucleation on same-charge substrates): however, such specific attractions are often thwarted if there is an overall electrostatic repulsion. Therefore, overall it appears that oppositely charged surfaces were most effective for protein nucleation. Although electrostatic attraction is not the overriding requirement for nucleant effectiveness, avoiding electrostatic repulsion is generally advantageous.

In addition, the presence of polar functional groups clearly offers a benefit compared to the unfunctionalised hydrophobic carbons which are generally all ineffective. A degree of surface hydrophilicity is required to encourage wetting of the solid nucleant surface by the condition and association with the proteins. Generally, nucleants with more hydrophilic moieties on the surface, are the most successful. The high degree (>10 μmol m^−2^) of polar functionalisation on CNM 8 and CNM 18, through acid oxidation, is consistent with the trends in the less heavily functionalised materials (<10 μmol m^−2^), but more effective for protein nucleation. To assess the possible effects of different specific functional groups, the MWNT(A) series was functionalised with a broad range of chemical species to a roughly similar extent (see Table 2 for surface group concentration). This series is intended to provide similar physical characteristics in terms of dimensions, packing and pore size, since the MWNTs are relatively rigid, and the gas phase functionalisation used does not change the length or carbon framework, and minimises capillary effects. Nevertheless, some changes in pore structure may occur during washing or due to secondary reactions; for example, the AA sample appears to undergo a cross-linking process, removing its dispersibility in water, and reducing its effectiveness as a nucleant. Note in these samples, the degree of polymerisation is only a few monomer units, creating short functionalised loops on the CNM surface. The most effective chemical group was MTEMA (CNM 6), followed equally by DMAEMA, MAA, DMAA and MeVP, representing amine, acid, acrylamide, and ammonium groups respectively, the majority of which are grafted with <10 wt% of oligo/polymer^20^,^21^. The effectiveness of the water compatibilising groups is expected, but the relative success of the mildly positively charged CNM 6 (thioether/ester) is less expected and may indicate additional specific interactions with the proteins; some oxide groups may also remain, in addition to the grafted species, and may generate mixed character surface. The different chemistry used to graft the mPEG functionality allowed very much higher molecular weight to be introduced (5 K ~ 112 monomer repeats), more similar to the PEG in the condition. In the case of CB (CNM 20), the effect of mPEG grafting was very significant, creating the most successful nucleant from the completely ineffective original ungrafted material (CNM 19). However, the related MWNT(A)-PEGMA was largely ineffective. Notably, the best solid nucleant, CNM 20, satisfies the requirement for a hydrophilic, negatively charged surface, but has a characteristically different physical structure.

### Porosity and curvature effects

The two most effective nucleants, CNMs 20 and 18 are both porous and have nanoscale roughness. It is possible that the intrinsic rigid pore structure of CNM 20 is maintained during work-up. Since GO (CNM 18) is highly hydrophilic, it continues to disperse easily in water (which was observed in the protein crystallization drops), and the effective surface area in solution is expected to be high; however, substantial restacking during drying means that the measured specific surface area of dry GO is not dramatically increased (~ 60 m^2^ g^−1^). The relative success of nucleants CNM 6 and CNM 8 may be attributed to the porous networks of functionalised MWNTs that form (specific surface area ~180 m^2^g^−1^, and ~ 191 m^2^g^−1^, respectively^33^); the most successful nucleant, CNM 20 (functionalised CB) had a similar surface area, around 217 m^2^g^−1^. In general, functionalised MWNT(B) materials were not effective as nucleants, due to i) a lower specific surface area (an order of magnitude lower) than the other nanomaterials and ii) low porosity with limited pore size distributions and large nanotube diameters. Note that the MWNTs functionalised by the gas phase process are not opened, as there is no etching of the carbon framework; thus these MWNTs remain closed, as original synthesised by CVD, and the active pores are the interparticle spaces. The acid oxidised MWNTs additionally offer a small internal pore, although the relative availability is very low, and the external pore network is still likely to dominate.

From specific surface area measurements, the average pore size for all the most successful nucleants, CNM 20, CNM 18, CNM 8 and CNM 6, is between 10 and 15 nm. It is likely that this pore size range stabilizes nuclei containing a small number of protein molecules; the average protein hydrodynamic diameters of lysozyme^34^, trypsin^35^ and thaumatin^36^ are 1.8 nm, 1.9 nm and 3 nm, respectively. The low rate of success in crystallizing catalase with any of the 20 nucleants, may be partly attributed to the large size of catalase^37^ (average hydrodynamic diameter 10.2 nm) compared to the average pore size of the nucleants. Although, the amorphous nature of carbon black makes it a low density and intrinsically porous material, the combination with PEG functionalisation is required to make a potent nucleant.

Although the precise mechanism of heterogeneous nucleation on porous substrates remains to be proven, indirect evidence and modelling studies can be used to make informed conjectures^3^,^11^,^12^,^13^,^17^. The fact that pore size is a crucial parameter in nucleant effectiveness and selectivity^3^,^17^ indicates that nucleation most likely takes place inside the pore, and the post-critical crystal then grows out of it. The useful pores are therefore not buried inside the bulk of the material but are located at its surface^12^,^13^. The active pores in the CNM samples are available at the surface as pockets and furrows formed by the networks of entangled nanotubes^17^ or troughs and depressions on the rough surface^32^.

Furthermore, there is no indication, either visual or by any anomalies in the X-ray diffraction, that crystals nucleated on porous substrates are qualitatively different or display any kind of inclusions that would show incorporation of the heterologous material into the crystal bulk. The only differences are in the size and diffraction quality, as expected when such crystals grow deep into the metastable zone, with respect to crystals grown conventionally.

## Conclusions

This study provides a first large-scale systematic comparison of a series of carbon nanomaterials, with different surface functionalisation, in terms of their effectiveness in inducing protein crystal nucleation. A comparative analysis highlights several parameters which are thought to influence nucleation-induction properties. It appears that porosity and/or roughness of a surface with a pore size distribution at the scale of the protein molecule (2–10 nm) is an important parameter in determining the success of a substance as a heterogeneous nucleant of macromolecular crystals. This observation is in agreement with results reported by our and other groups over many years, with different materials sharing this characteristic^2^,^3^,^4^,^5^,^6^,^11^,^16^,^27^. In addition, a high accessible surface area maximises the efficiency of a given quantity of nucleant. The surface chemistry should be modified to be hydrophilic, or at least amphiphilic, generally with an opposite charge to the protein in question, although specific protein interactions may also be important. Functionalised CNMs may be particularly attractive as their reactions tend to create heterogeneous surfaces, as quantified by inverse gas chromatography^38^, the variation in surface character may play an important role in providing at least one optimal site from which proteins can nucleate. This combination of surface area, porosity and surface heterogeneity (created by topography and chemistry) makes carbon black nanoparticles functionalised with mPEG 5 K (CNM 20) a highly effective nucleant, even as a dried powder.

This material induced nucleation at unequivocally metastable conditions, for four out of the five proteins used. Most importantly, CNM 20 rescued a target protein, namely RoAb13, which might otherwise have been discarded. In this instance, CNM 20 was more effective than the other most widely successful nucleants. The advantage of the carbon black nanoparticles functionalised with mPEG 5 K over the current known nucleants is that it is simple to manufacture and its effectiveness is very reproducible. Furthermore, there is a broad class of carbon black nanoparticles available, beyond the material used in this work, with different primary particle size, degree of agglomeration, and hence different sized cavities. These materials could also be functionalised either by thermal activation or reduction, following the methods described in this work, and readily used as nucleants.

## Experimental Section

Two types of MWNTs were used in this study for further functionalisation. MWNTs with diameters ~ 10 nm were obtained from Arkema SA (Lacq-Mourenx, France), hereafter referred to as MWNT(A). MWNTs with larger diameters (80–100 nm) grown in house via injection chemical vapour deposition were also used (MWNT(B))^39^ Graphene oxide (GO) was purchased from Nanoinnova Technologies, S.L. with a lateral average size and thickness in the range of 1–4 μm and 0.7–1.2 nm, respectively. Carbon black nanoparticles (Printex 90, primary particle diameter 21.1 nm, ± 6.2 nm) were purchased from Degussa-Hüls (Frankfurt, Germany).

Monomers for functionalisation: acrylic acid (AA, 99%), acrylonitrile (AN, ≥ 99%), 2-(dimethylamino) ethyl methacrylate (DMEAMA, 98%), methyl methacrylate (MMA, > 98.5%), 2-(methylthio) ethyl methacrylate (MTEMA, 96%), N,N-dimethylacrylamide (DMAA, 99%), 4-vinyl pyridine (4-VP, 95%), poly(ethylene glycol) methacrylate (PEGMA, average M_W_ 530), were purchased from Sigma-Aldrich for CNM functionalisation. For the synthesis of N-methyl-4-vinyl pyridine (MeVP), the 4-VP-CNMs (20 mg) were dispersed in 10 ml methanol (99.8%, Sigma-Aldrich) by bath sonication for 5 min; iodomethane (IMe) (3.12 ml, 50.0 mmol) was added dropwise, and the reaction mixture was heated to 60 °C overnight under N_2_ atmosphere^21^. The mixture was cooled to room temperature and filtered through a 0.45 mm PTFE membrane. The CNMs were washed with 3 × 30 ml of ethanol, then dispersed in 30 ml of ethanol and bath sonicated for 15 min. The filtration-sonication cycle was repeated three times in order to remove any physically absorbed reactants. To obtain methacrylic acid (MAA) functionalised materials, LiOH (40 mg) was dissolved in 20 mL 10:1 v/v THF/water cosolvent before adding 20 mg of MMA functionalised CNMs. The reaction mixture was bath sonicated for 5 min to obtain a good dispersion, and then stirred at room temperature overnight. Subsequently, 37% hydrochloric acid (HCl, AnalaR grade, BDH) was added drop-wise until the solution reached pH 2. The mixture was stirred for another 12 h, then filtered through a 0.45 μm PTFE membrane, and washed with water (3 × 30 mL). The filtration-sonication cycle was repeated three times in order to remove any remaining salt and acid. Before use, monomers used as received were passed through a chromatographic column consisting of neutral and basic aluminium oxide powders (aluminium oxide 90 (0.063–0.200 mm), activity stage I for column chromatography, Merck Millipore, Germany) and further degassed by bubbling N_2_ gas for 30 min, in order to remove radical inhibitors and oxygen.

### Functionalisation by thermochemical grafting

The thermal activation process was carried out in a custom-made 30 mm diameter quartz tube attached to a sample flask, and the whole setup was connected to a vacuum system. In a typical experiment,100 mg of MWNTs were heated to 1000 °C at a constant ramping rate of 10 °C/min under vacuum (~ 5 × 10^−4^ mbar), in a three zone tube furnace (PTF 12/38/500, Lenton Ltd, UK) and held at the activation temperature for 2 h. After the activation step, the quartz tube was slowly removed from the heating zone and allowed to cool to room temperature under vacuum. The MWNTs were then transferred to the connected round bottom flask by gravity. 8 ml of the reactant was then injected into the flask containing the thermally-activated MWNTs. The reaction mixture was stirred at room temperature overnight. The unreacted reactant was removed via filtration through a 0.45 μm pore size polytetrafluoroethylene (PTFE) membrane (Whatman, UK) under vacuum. The product was thoroughly washed with 3 × 90 ml of washing solvent, then dispersed in 90 ml of solvent and bath sonicated (USC300T, 45 kHz, 80 W, VWR International, USA) for 15 min. Transmission electron micrographs were obtained using JEOL 2000 transmission electron microscope operated at an accelerating voltage of 200 kV. The measurements of adsorption and desorption isotherms of nitrogen at 77 K were carried out on 20–50 mg of CNMs using Micromeritics ASAP 2010 apparatus. Specific surface areas were calculated according to the Brunauer, Emmett and Teller (BET) equation from the adsorption isotherms in the relative pressure range of 0.05–0.20 *p/p*_*0*_. The thermally oxidised samples (CNM 3 – CNM 6) were heated in air at 400 °C to remove any low temperature burning amorphous carbon before further monomer functionalisation by thermochemical grafting^40^. CNM 8 is the only acid oxidised sample; for this sample, the as-received MWNTs were refluxed in concentrated sulphuric and nitric acid^33^, this method introduced oxygen containing functionalities and is a damaging process to the nanotube structure.

### Functionalisation by reduction of CNMs

Reduction chemistry was utilised to produce carbon black by Na/naphthalene in dimethylacetamide (DMAc) for functionalisation with poly(ethylene glycol) methyl ether (mPEG). DMAc, sodium (99.95%, ingot, No. 262714), naphthalene (99%) were obtained from Sigma-Aldrich and used as received. mPEG, of mean M_W_ 5000, was purchased from Sigma-Aldrich and brominated by the aldol reaction. Poly(ethylene glycol) methyl ether (mPEG) M_W_ 5000 and 1 eq. of triphenylphosphine (PPh_3_) and 4 eq. of tetrabromomethane (CBr_4_) were refluxed in dry dichloromethane (DCM) for 24 hr. under N_2_. After removal of remaining DCM, mPEG-Br was precipitated into cold ether (−78 °C), the mPEG-Br was then filtered using a Buchner funnel and washed with cold ether.

A typical protocol for the preparation of reduced CB involved heating to 400 °C under vacuum (<10^−2^ mbar) for 1 hr. Naphthalene was dried overnight in a vacuum oven with phosphorous pentoxide before transferring to a N_2_ filled glove box. 1 mmol of sodium and 1 mmol of naphthalene were added to 10 ml of degassed THF and stirred for 1 day. A dark green colour was observed. 1 ml of the Na/naphthalene solution was added to dry CB and more degassed THF was added to adjust the sodium concentration (0.1 M). The solution was sonicated for 15 minutes and stirred for 1 day before adding mPEG-Br and stirring for an additional 24 hours. The solution was removed from the glove box after 24 hr. and quenched with dry O_2_. After bubbling dry O_2_ into the solution, the solution was stirred overnight under dry O_2_ for oxidation of any remaining charge on the functionalised CB^24^.

The as-received materials were also tested as nucleants for control purposes. No further functionalisation was performed on GO. The nanocarbon substrates, the chemical groups with which they were functionalised and the full list of nucleant candidates used in this study are shown in Tables 1 and 2.

### Crystallization experiments

Thaumatin, lysozyme, trypsin and catalase were obtained as highly pure lyophilised powders from Sigma-Aldrich, UK. Thaumatin (T-7638) from *Thaumatococcus daniellii* was prepared in distilled water at 30 mg/ml; lysozyme (L-6876) from chicken egg-white was prepared in 50 mM sodium acetate at concentrations of 20 and 40 mg/ml; trypsin (T9201) from porcine pancreas was prepared in 10 mg/ml benzamidine hydrochloride, 10 mM calcium chloride and 20 mM HEPES (pH 7.0) at 60 mg/ml; and catalase (C-9322) from bovine liver was prepared in 10 mM HEPES buffer (pH 7.0) at a concentration of 15 mg/ml. The anti-CCR5 Fab fragment RoAb13 was given by Professor Benny Chain (University College London) and was prepared in 0.1 M sodium chloride and 20 mM HEPES (pH 7.0) at 10 mg/ml.

All reagents were of analytical grade, obtained commercially. All solutions were freshly prepared, using milli-Q water (Merck Millipore, USA). Salt and buffer stock solutions were kept at room temperature for the duration of the study. Polyethylene glycols (PEG) of various mean molecular weights, namely 2000 methyl ether (84797, Sigma-Aldrich, UK); 3350 (88276, FlukaBiochemika); 8000 (P-4463, Sigma-Aldrich, UK) were used to make solutions kept at 4° C. Paraffin oil (294365 H, BDH, UK) was used for the microbatch trials.

The nucleants were inserted into hanging drop vapour diffusion trials using 15-well EasyXtal plates and EasyXtal X-seal screw lids (Qiagen, UK). The drops consisted of 1 μL protein solution to which 1 μL of reservoir solution was added. Each nucleant-protein combination was set up in triplicate for each tested condition (see Results), with at least one corresponding control without nucleant. The controls were dispensed on the same lids as their respective test drops and therefore sealed over the same well. Microbatch trials^41^ performed with lysozyme also consisted of 2 μL drops, dispensed under paraffin oil in Terazaki plates. In this case, control drops were dispensed from the same solution in different wells.

For each protein, the protein concentration, buffer pH and concentration, and (in the cases of trypsin and RoAb13) additive type and concentration were kept fixed, as shown below. The concentration of precipitant was systematically varied in order to access the metastable zone. The conditions were as follows:

*Thaumatin*: 0.1 M Bis-tris propane at pH 6.8 and 0.1–1 M Na/K tartrate (NaKT)

*Lysozyme*: 0.1 M sodium acetate at pH 4.5 and 0.5–1.5 M NaCI

*Trypsin*: 0.2 M ammonium sulphate, 0.1 M cacodylate at pH 6.5 and 5–30%(w/v) PEG 8000

*Catalase*: 0.1 M tri-ammonium citrate at pH 6.8 and 10–20%(w/v) PEG 3350

*RoAb13*: 10 mM nickel chloride, 0.1 M Tris at pH 8.5 and 16%–20%(w/v) mPEG2K.

## Additional Information

**How to cite this article**: Govada, L. *et al*. Exploring Carbon Nanomaterial Diversity for Nucleation of Protein Crystals. *Sci. Rep*. **6**, 20053; doi: 10.1038/srep20053 (2016).

## Figures and Tables

**Figure 1:**
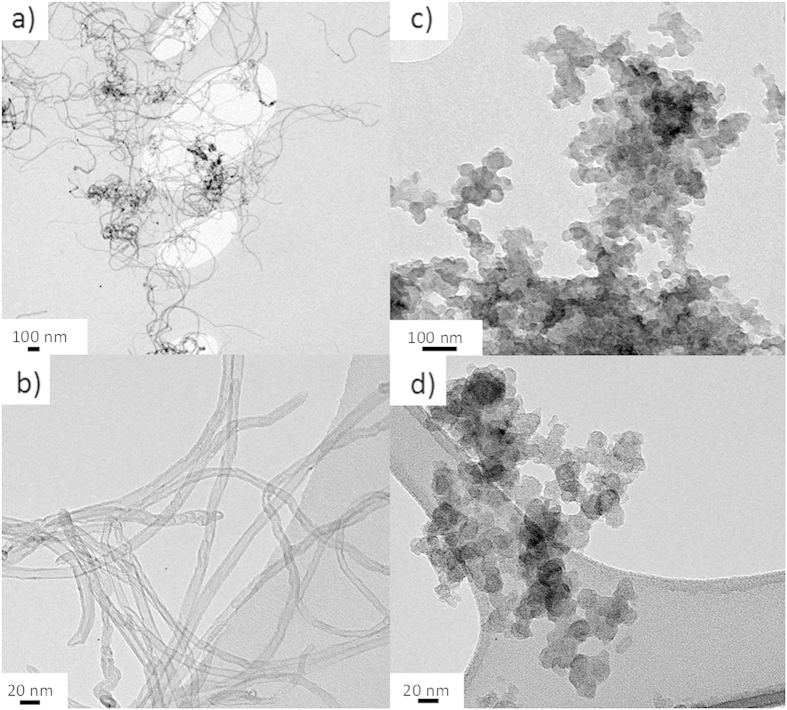
TEM images of carbon nanomaterials used: (**a**) functionalised MWNTs of individualised and bundling nanotubes; (**b**) higher resolution image of the network formation of MWNTs; (**c,d**) carbon black nanoparticles functionalised with PEG forming agglomerates of nanoparticles between 1 μm and 100 nm.

**Table 1:** Nanocarbon substrates.

Abbr.	Carbon material	Description	BET surface area beforefunctionalisation (m^2^g^−1^)	BET surface area postfunctionalisation (m^2^g^−1^)
MWNT(A)	multi-wall carbon nanotubes	CVD grown commercial Arkema^®^ CNTs, D approx. 10 nm and several microns in length	220	180
MWNT(B)	multi-wall carbon nanotubes	Injection CVD grown carbon nanotubes D approx. 100 nm and several tens of microns in length	30	<30
GO	graphene oxide	Heavily-oxidised, hydrophilic graphene	60	—
CB	carbon black	Common form of amorphous carbon, agglomerate size 100–500 nm, average primary particle size 10 nm.	270	220

**Table 2:**
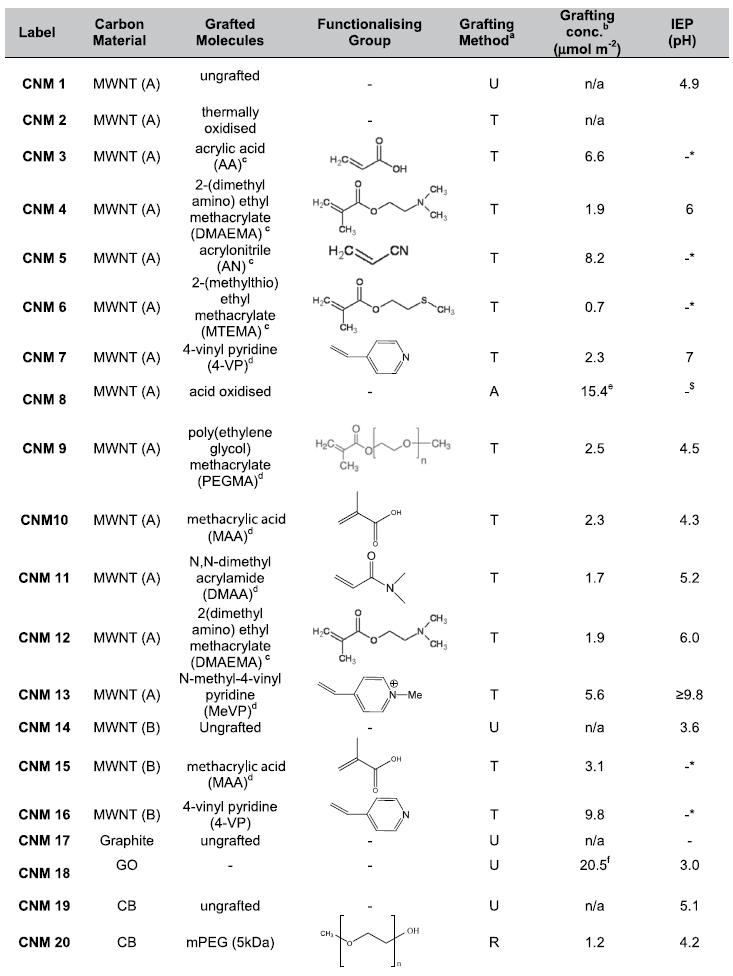
A catalogue of as received (commercial/unmodified materials) and functionalised carbon nanomaterials tested.

^a^grafting methods U – ungrafted T – thermochemical R – reduction

^b^calculated from polymer grafting ratio and BET measurements

^c^see ref. 20

^d^see ref. 21

^e^see ref. 33

^f^determined by titration * not measured due to low solubility in water ^$^ no IEP the material is negative across pH range

**Table 3:**
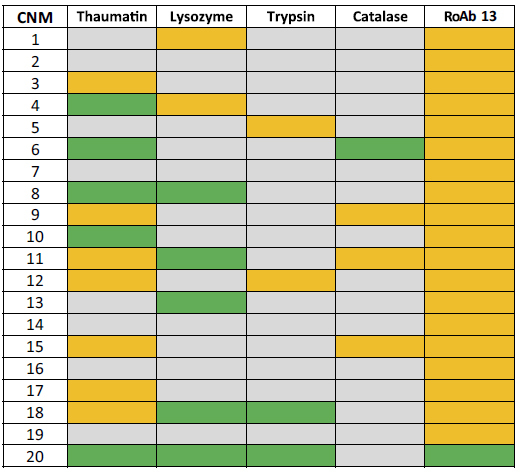
Carbon Nanomaterials (CNM) nucleation test results.

Nucleants were added to trials as small solid flakes. Key: 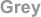 colour indicate clear drops where no crystal growth was seen. 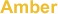 indicates borderline conditions, namely crystals growing at the cusp of metastable and nucleation regions. 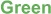 represents crystals growing deep in the metastable conditions.
